# Identification of Circadian Clock Homologs and Their Rhythmic Expression Differences Among Mating-Type Strains in *Morchella sextelata*

**DOI:** 10.3390/jof12060404

**Published:** 2026-06-02

**Authors:** Meng-Qian Chen, Jun-Xi Liu, Jia Ling, Xi-Hui Du

**Affiliations:** College of Life Sciences, Chongqing Normal University, Chongqing 401331, China; cmq1822461288@163.com (M.-Q.C.); shimizu05@163.com (J.-X.L.); lj2585758921@163.com (J.L.)

**Keywords:** circadian clock, frequency, clock-controlled genes, homolog, co-culture, crossed conidition, mating-type

## Abstract

The circadian clock is a widespread rhythmic phenomenon across organisms, characterized by distinct gene expression patterns and behaviors at specific times of the day. Extensive genetic studies in the model fungus *Neurospora crassa* have yielded critical insights into the components and molecular mechanisms of circadian oscillators. However, these understandings remain absent across fungal lineages, especially from edible mushrooms. Morels (*Morchella* spp.) are well-recognized edible ascomycetes of considerable economic value and are partially artificially cultivated, but their biological characteristics are poorly understood. Investigating the presence of their circadian clock components, as well as the molecular underpinnings of circadian rhythms, holds important biological implications. In this study, we firstly performed a genomic search for homologs of known circadian clock genes in *Morchella sextelata*. Homologs of seven circadian clock genes, including *wc-1*, *wc-2*, *fwd-1*, *frh*, *frq*, and two additional clock-controlled genes, were identified, indicating the components necessary for the operation of a FWC oscillator contained in *M. sextelata*. Then, using reverse transcription quantitative PCR (RT-qPCR), the expression profiles of these seven circadian clock-related genes and four mating-type genes were examined in RNA samples which were extracted from mycelia of *MAT1-1*, *MAT1-2* and *MAT1-1* × *MAT1-2* co-culture/crossed condition during conidiation under in vitro cultivation across one day. The expression levels of seven circadian clock genes and four mating-type genes displayed similar time-of-day-specific rhythmic patterns, yet remained consistently distinct across the mating-type strains and their co-culture/crossed condition, indicating a potential correlation between circadian clock and mating-type loci. Collectively, these results suggest that *M. sextelata* harbors conserved circadian clock-related homologs and displays mating-type-associated temporal expression differences under the tested conidiation conditions, offering a novel perspective for exploring the potential link between clock-related regulation and mating-type background in the future.

## 1. Introduction

Circadian clocks are endogenous timekeeping systems that synchronize with environmental cues, and play a vital role in coordinating gene expression, physiology, and behaviors across the tree of life in a ~24 h cyclic oscillation [1,2,3,4]. The circadian clock is evolutionarily conserved across eukaryotes and relies on a transcription–translation feedback loop (TTFL), comprising positive regulators (WC-1/WC-2 in *Neurospora crassa*, CYC/CLK in *Drosophila*, and BMAL1/CLOCK in mammals) and negative components (FRQ, TIM/PER, and CRY/PER in the corresponding organisms, respectively) [5,6,7,8,9]. In fungi, *N. crassa* is the most extensively characterized model fungus for circadian rhythm research, with its clock system widely documented to regulate key biological processes, including conidiation, sexual reproduction, hyphal growth rate, and aerial hyphae formation [10,11,12]. Its circadian clock system is centered on the FRQ/WCC oscillator [13], which comprises core molecular components including FRQ (FREQUENCY), FRH (FRQ-interacting RNA helicase), WC-1 (White Collar-1), WC-2 (White Collar-2) and FWD-1 (F-box/WD40 repeat-containing protein-1) [14,15]. Besides the core molecular components, there is also a group of well-described clock-controlled genes (*ccgs*) [16,17], which are downstream target genes of the circadian oscillator [18,19].

The fungal kingdom is estimated to comprise up to 5.1 million potential species [20]. However, except for the model fungus *N. crassa* [2], studies on circadian clocks, related phenomena, and oscillator components in other fungi are largely limited, especially in edible fungi. One effective approach to elucidate circadian rhythms in other fungi is to examine the distribution of the core clock genes from *N. crassa* in their genomes and characterize their expression patterns [19,21,22,23]. *Morchella*, a genus of edible and medicinal fungi, has garnered increasing attention due to its artificial cultivation and substantial economic value [24,25,26,27]. Given that the yield and quality of *Morchella* fruiting bodies are tightly linked to the coordination of developmental events (e.g., mycelial growth, conidiation, primordium formation and fruiting body maturation) with environmental cycles, investigating its circadian clock is theoretically and practically relevant for better understanding its life cycle. Furthermore, despite the circadian clock being well-documented in model fungi, whether circadian rhythms are modulated in the co-culture/crossed condition between *MAT1-1* and *MAT1-2* strains remains unexplored. Likewise, comparisons of circadian rhythmicity among strains with different mating types remain poorly investigated. *Morchella* mainly displays a heterothallic mating system, in which the *MAT1-1* idiomorph harbors *MAT1-1-1*, *MAT1-1-10*, and *MAT1-1-11*, whereas the *MAT1-2* idiomorph contains only *MAT1-2-1* [24,26]. Comparative studies on the circadian clock gene expression among its different mating-type and co-culture/crossed conditions are helpful to uncover the rhythmic difference between mating types.

In this study, we identified putative homologs of known fungal circadian clock-related genes in the genome of *M. sextelata* and examined the temporal expression patterns of selected clock-related homologs and mating-type genes in *MAT1-1*, *MAT1-2* and *MAT1-1* × *MAT1-2* co-culture during conidiation. By comparing expression profiles across these mating-type backgrounds, we aimed to determine whether clock-related homologs and mating-type genes exhibit time-of-day-dependent transcriptional differences. These analyses provide an initial framework for understanding clock-related transcriptional dynamics in *M. sextelata* and for exploring their potential association with mating-type regulation.

## 2. Materials and Methods

### 2.1. Strains Used in This Study

The wild fruiting body of *Morchella sextelata*, collected in China, was used in this study, with species identification confirmed by phylogenetic analysis of the ITS dataset. Its single mating-type strains with either *MAT1-1* or *MAT1-2* were obtained by isolating single ascospores which were detected using mating-type genes. The *MAT1-1* and *MAT1-2* isolates were maintained separately for single-strain experiments and were also used to establish the *MAT1-1* × *MAT1-2* co-culture/crossed condition described below.

### 2.2. Growth Conditions and Sampling

For analysis of circadian rhythms and detection of mating-type-associated differences, the *MAT1-1* strain, *MAT1-2* strain and *MAT1-1* × *MAT1-2* co-culture/crossed condition with 24 replicates for each treatment were inoculated on Murashige and Skoog medium (MS; Solarbio Science & Technology Co., Ltd., Beijing, China) and then cultivated for 24 days to induce conidiation under the following conditions: placing these MS plates on a clean bench at free-running indoor temperature in the lab with windows open from December to January by mimicking natural growth conditions with light from the lab windows and occasional lab illumination. For the *MAT1-1* × *MAT1-2* co-culture/crossed condition, *MAT1-1* and *MAT1-2* isolates were inoculated on the same MS plate to allow mycelial contact. Dense conidial production was mainly observed in the contact region between the two mating-type isolates. At each sampling time point, mycelial tissues from the co-culture plate, including the contact region, were harvested for RNA extraction. Therefore, this treatment contained mycelial material from both *MAT1-1* and *MAT1-2* mycelia and their contact region. Because heterokaryon formation at the contact region could not be directly verified in the present study, this treatment is referred to as the *MAT1-1* × *MAT1-2* co-culture/crossed condition. On the 24th day, three replicates of each treatment were harvested every 3 h over 24 h. Finally, a total of 72 samples were harvested and obtained for RNA extraction and RT-qPCR, including three replicates of each treatment at 0 h, 3 h, 6 h, 9 h, 12 h, 15 h, 18 h, and 21 h, respectively. Zeitgeber time (ZT) denotes an exogenous temporal framework that anchors biological rhythms to a principal environmental synchronizer (Zeitgeber, a German term literally meaning “time giver”). Herein, ZT labels are used to denote chronological sampling points under the tested culture condition, such as 3:00 p.m. corresponding to ZT15:00.

### 2.3. Homolog Identification of Circadian Clock Genes and Acquisition of Mating-Type Genes in M. sextelata

Seventeen clock-related genes, including four core circadian clock genes (*wc-1*, *wc-2*, *frh*, *frq* from *N. crassa*, *Aspergillus chevalieri* and *A. melleus*) [11], and 13 additional clock-associated or clock-controlled genes (*fwd-1*, *pre-1*, *pre-2*, *mfa-1* and seven *ccgs* from *N. crassa* and two *ccgs* from *Lachnellula hyalina*) [11,28,29,30,31] were used as queries to search for their putative homologs in the *M. sextelata* genomic databases in NCBI using BLASTX [32]. Details of all query circadian clock genes used are listed in Table 1. Orthology of the *M. sextelata* genes was assessed and potential homologs in *M. sextelata* were retrieved using an E value less than 1 × 10^−10^, species identity over 30% and coverage over 25% as selection criteria. Four mating-type genes, including *MAT1-1-1*, *MAT1-1-10*, *MAT1-1-11* and *MAT1-2-1* were acquired from the publicly available reference genome of *M. sextelata* in the NCBI database (GenBank accession number: GCA_020137385.1) and via PCR amplification.

Primers for the finally selected homologs of circadian clock genes and mating-type genes were designed using NCBI Primer-BLAST (National Center for Biotechnology Information, Bethesda, MD, USA; https://www.ncbi.nlm.nih.gov/tools/primer-blast/, accessed on 1 May 2026) [33] with the following criteria: length: 18–22 bp; amplicon size: 80–200 bp; melting temperature (Tm): 50–60 °C; and GC content: 40–60%. The specificity of all primers designed was further validated using BLAST and the NCBI Primer-BLAST tool.

### 2.4. RNA Extraction and RT-qPCR

Samples, including hyphae and conidial tissues for each, were ground in liquid nitrogen and total RNA was extracted using TRIzol reagent (Accurate Biology Co., Ltd., Changsha, China) according to the manufacturer’s instructions. The concentration of total RNA was measured with a Nanodrop Analyzer (Allsheng Instruments Co., Ltd., Hangzhou, China). To assess the integrity of the RNA, the total RNA were run on a denaturing gel to check for the presence of clear 28S and 18S rRNA bands at a 2:1 ratio. Then, the total RNA of each group was used as the template for reverse transcription with the Evo M-MLV reverse transcription kit (Accurate Biology Co., Ltd., Changsha, China).

Real-time quantitative PCR (RT-qPCR) was used to quantify gene expression of seven circadian clock-related homologs and four mating-type genes in *M. sextelata*, elucidating 24 h regulatory trends of 11 genes during conidiation. The primers of 11 targeted genes for RT-qPCR are detailed in Table 2. RT-qPCR was conducted using a CFX Connect real-time PCR detection system (Bio-Rad, Hercules, CA, USA) at 95 °C for 2 min, and then 40 cycles at 95 °C for 10 s followed by 52 °C for 15 s, and 72 °C for 15 s with *actin1* selected as the normalization reference after candidate reference-gene validation (internal control) in this study. The instrument default was set to melt-curve analysis. The total volume of the reaction system was 20 μL, including 10 μL of 2× SYBR^®^ green premix ProTaq HS kit (Accurate Biology Co., Ltd., Changsha, China), 0.6 μL each of positive and negative primers, 1 μL of cDNA and 7.8 μL of RNase-free ddH_2_O. The method of 2^−ΔΔCt^ was used to calculate the relative expression ratios of target genes [34]. Independent assays were performed three times.

### 2.5. Reference-Gene Validation for RT-qPCR Normalization

To validate the suitability of reference genes for RT-qPCR normalization under the circadian time-series sampling condition, three candidate reference genes, *actin1*, *elongation factor 1-α* and *β-tubulin* were evaluated in this study using primers reported in [35]. Raw Ct values of these candidate genes across all tested samples were used for stability analysis with the BestKeeper algorithm. The stability of each candidate reference gene was assessed based on standard deviation (SD), coefficient of variation (CV) and correlation with the BestKeeper index. The gene showing the highest expression stability was selected as the internal reference for normalization of target gene expression across circadian time during conidiation of *M. sextelata*.

### 2.6. Statistical Analysis

Statistical analyses of the rhythmic expression profiles of the 11 investigated genes across time points were performed using SPSS 27.0, employing one-way ANOVA, with a significance level set at *p* < 0.05. The GraphPad Prism version 10.3.0 was utilized for data visualization.

## 3. Results

### 3.1. Conidiation Comparison of MAT1-1, MAT1-2 and MAT1-1 × MAT1-2 Co-Culture

**Figure 1:**
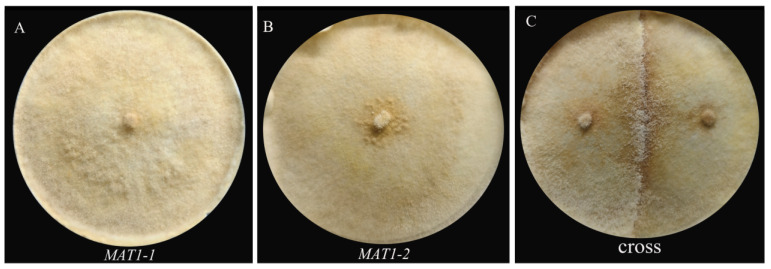
Colony morphology of the *MAT1-1* (**A**), *MAT1-2* (**B**) and *MAT1-1* × *MAT1-2* co-culture/crossed condition (**C**) strains of *Morchella sextelata* sampled for assessing rhythmic oscillations in gene expression of seven clock homologous genes and four mating-type genes.

We examined the conidial growth of cultures grown on MS medium over a 24-day period. A marked difference between single *MAT* cultures and the *MAT1-1* × *MAT1-2* co-culture/crossed condition was observed. *MAT1-1* and *MAT1-2* strains displayed only sporadically scattered conidia on their respective media. At the junction between *MAT1-1* and *MAT1-2* in the co-culture/crossed condition, conidia clustered densely in their contacted region (Figure 1). Because heterokaryon formation could not be experimentally verified, this phenotype is interpreted as a co-culture-associated conidiation pattern rather than direct evidence of a genetically confirmed heterokaryotic strain.

### 3.2. Identification of Circadian Clock Gene Homologs in M. sextelata

In order to investigate the presence of circadian clock-associated genes in *M. sextelata*, we examined its genome for homologs of the four core circadian clock genes (*frq*, *wc-1*, *wc-2*, *frh*), nine clock-controlled output genes (*ccgs*) and four additional clock-controlled genes (*mfa-1*, *pre-1*, *pre-2*, *fwd-1*), which were identified from *N. crassa*, *A. chevalieri*, *A. melleus* and *L. hyalina*, respectively [11,28,29,30,31]. Then, homologs of four core circadian clock genes (*wc-1*, *wc-2*, *frh*, *frq*) and one clock gene (*fwd-1*) were identified in the *M. sextelata* genome, sharing 30.85–61.04% sequence identity with queries (Table 1). Among the nine *ccgs* of *N. crassa* and *L. hyalina*, only homologs of five (*ccg-8*, *ccg-9*, *ccg-15*, *ccg-16*, *ccg-14*) were identified in *M. sextelata*, sharing 33.33–51.72% sequence identity with queries. Due to low coverage of homologs of *ccg-15*, *ccg-16* and *ccg-14* to those of *N. crassa*, the three homologs were not further investigated in this study. Homologs of *ccg-1*, *ccg-4*, *ccg-6*, *ccg-13*, *mfa-1*, *pre-1*, and *pre-2* have not been identified in the genome of *M. sextelata*, with nothing matched. The homologs of circadian clock genes in *M. sextelata* are summarized in Table 1. The results indicated that *M. sextelata* contain *frq*, *frh*, *wc-1*, *wc-2*, and *fwd-1* homologs (Table 1), indicating that this species contains the components necessary for a FWC oscillator as is found in *N. crassa*. It also contain homologs for *ccg-8* and *ccg-9* of the output pathway of the FWC oscillator in *N. crassa*.

### 3.3. Validation of Candidate Reference Genes for RT-qPCR Normalization

To ensure the reliability of RT-qPCR normalization under the present time-series sampling condition, three candidate reference genes, *actin1*, *elongation factor 1-α* and *β-tubulin*, were evaluated using the BestKeeper algorithm based on their raw Ct values. All three candidate reference genes showed SD values below 1, indicating acceptable expression stability under the tested experimental conditions. Among the three candidates, *actin1* showed the lowest SD value and a high correlation with the BestKeeper index. Although *elongation factor 1-α* displayed a slightly higher correlation coefficient, *actin1* exhibited lower Ct variation and a moderate expression level. Therefore, *actin1* was selected as the internal reference gene for normalization of clock-related homologs and mating-type genes in the subsequent RT-qPCR analysis (Table 3).

### 3.4. Expression Patterns of Seven Circadian Gene Homologs in MAT1-1, MAT1-2 and MAT1-1 × MAT1-2 Co-Culture

To elucidate whether the seven circadian homologs (*frq*, *frh*, *fwd-1*, *wc-1*, *wc-2*, *ccg-8* and *ccg-9*) found in *M. sextelata* had rhythmic periodicity, we applied RT-qPCR and analyzed the expression patterns of them in the *MAT1-1*, *MAT1-2* and their co-culture/crossed conditions at eight time points of the conidiation stage during the ZT cycle of one day.

#### 3.4.1. Expression Patterns of Seven Circadian Gene Homologs in the *MAT1-1* Strain

**Figure 2:**
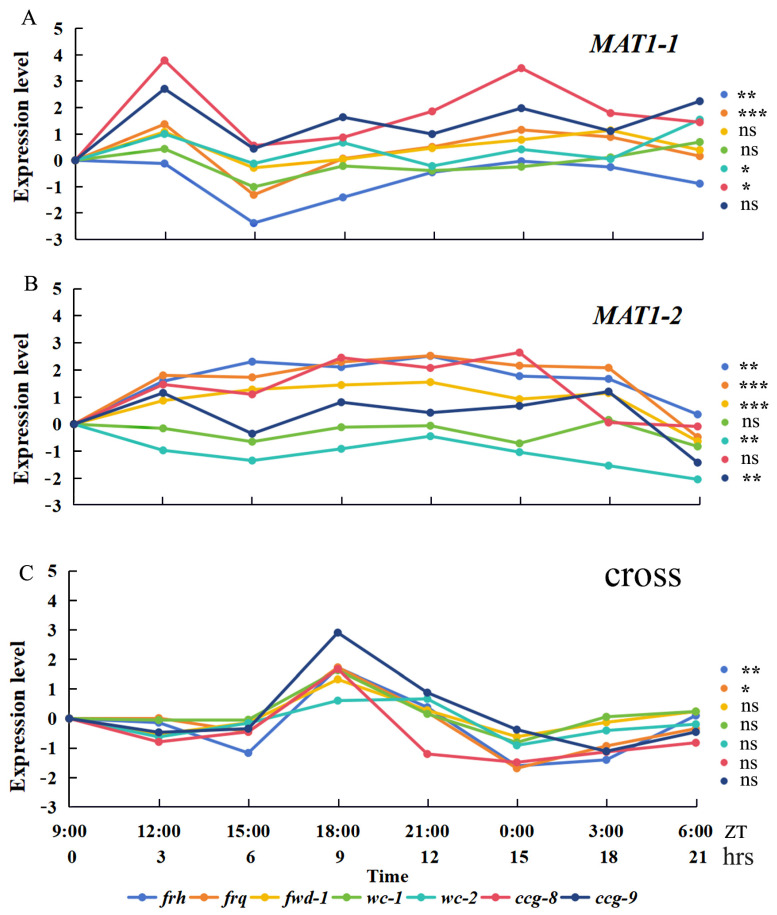
Rhythmical oscillations in gene expression of seven clock homologous genes (*frh*, *frq*, *fwd-1*, *wc-1*, *wc-2*, *ccg-8* and *ccg-9*) of *M. sextelata* in its *MAT1-1* (**A**), *MAT1-2* (**B**) and *MAT1-1* × *MAT1-2* co-culture/crossed condition (**C**) strains. Total RNA was isolated from each strain and harvested every 3 h for one day (x-axis). The expression levels of the seven homologous genes were quantified using the expression of the housekeeping *actin1* as a reference (y-axis). Each dot represents the mean of three biological replicates. Note that the transcript profiles of seven homologous genes oscillate in generally similar patterns in each strain, but are totally different among the three strains, reaching peak levels at different timepoints. Zeitgeber time (ZT) is depicted. *frh*: frequency-interacting RNA helicase; *frq*: frequency; *wc*: white collar; *ccg*: clock-controlled gene. Statistical significance was assessed by one-way ANOVA (*: *p* < 0.05; **: *p* < 0.01; ***: *p* < 0.001; ns = not significant).

All the seven circadian gene homologs had one high expression peak at ZT12:00 and one low expression trough at ZT15:00 (Figure 2A). After the peak at ZT12:00, the expression of *ccg-8*, *frh* and *frq* showed similar rhythmic fluctuation patterns with the second peak at ZT0:00, while *fwd-1* had the second peak at ZT3:00. In addition, the expression of *wc-1*, *wc-2* and *ccg-9* showed similar rhythmic fluctuation patterns with two or three peaks at ZT18:00, ZT0:00 and ZT6:00 after the peak at ZT12:00, respectively.

One-way ANOVA confirmed significant rhythmic variation in four of the seven genes, whereas expression changes in *fwd-1*, *wc-1*, and *ccg-9* did not reach statistical significance (Figure 2A). Notably, even these non-significant genes exhibited peak timing broadly similar to the significant ones, suggesting a coordinated expression program across all seven genes. In addition, the current expression patterns of seven circadian gene homologs in the *MAT1-1* strain indicate a potential rhythmic periodicity of 12 h. However, since this study only investigated a 24 h period, further research with a longer duration is needed.

#### 3.4.2. Expression Patterns of Seven Circadian Gene Homologs in the *MAT1-2* Strain

Notably, the expression patterns of seven circadian gene homologs in the *MAT1-2* strain were totally different from those in the *MAT1-1* strain, and did not exhibit clear regular rhythmic oscillation, instead presenting a single broad, gently rounded expression peak (Figure 2B). The expression levels of *ccg-8*, *frh*, *frq* and *fwd-1* were consistently higher than those of *wc-1*, *wc-2* and *ccg-9*. One-way ANOVA verified significant rhythmic variation in five of the seven genes, while the expression changes in *wc-1* and *ccg-8* were not statistically significant (Figure 2B). Nevertheless, genes that lacked significant rhythmicity still displayed broadly consistent peak phases with the significant genes. Rhythmic patterns indicate that these genes in the *MAT1-2* strain are likely under circadian regulation with an approximately 24 h period.

#### 3.4.3. Expression Patterns of Seven Circadian Gene Homologs in the *MAT1-1* × *MAT1-2* Co-Culture

Interestingly, the expression patterns of seven circadian gene homologs in the co-culture/crossed condition were totally different from those in both the *MAT1-1* strain and *MAT1-2* strain, but their expression demonstrated a surprisingly consistent rhythmic fluctuation pattern (Figure 2C). All the seven circadian gene homologs (*ccg-8*, *frh*, *frq*, *fwd-1*, *wc-1* and *ccg-9*) had the highest expression peak at ZT18:00, with an exception of *wc-2* at ZT 21:00. They also exhibited nearly identical expression levels at ZT18:00, except for *ccg-9*, which showed a significantly higher expression level. All the seven circadian gene homologs (*ccg-8*, *frh*, *frq*, *fwd-1*, *wc-1* and *ccg-9*) had the lowest expression at ZT0:00, with an exception of *ccg-9* at ZT3:00.

According to one-way ANOVA analysis, only *frh* and *frq* among the seven genes showed significant rhythmic expression changes in the co-culture/crossed condition (Figure 2C), while the other five genes did not exhibit statistically significant rhythmicity. Despite lacking statistical significance, these genes still showed comparable peak phases to the significant ones, supporting the presence of a unified expression profile across all seven genes. The circadian period of seven circadian gene homologs in the co-culture/crossed condition remains unclear, owing to the short duration of investigations into their expression patterns.

#### 3.4.4. Comparison of Expression Patterns Among *MAT1-1*, *MAT1-2* and *MAT1-1* × *MAT1-2* Co-Culture

The expression patterns of seven circadian gene homologs were totally different among *MAT1-1*, *MAT1-2* and their co-culture/crossed condition. Seven circadian gene homologs in the *MAT1-1* strain (Figure 2A) had similar expression pattern to some extent but still showed partial differences between the four gene homologs (*ccg-8*, *frh*, *frq*, *fwd-1*) and the other three (*wc-1*, *wc-2*, *ccg-9*). It is the same case in the *MAT1-2* strain, where the four gene homologs (*ccg-8*, *frh*, *frq*, *fwd-1*) and the other three (*wc-1*, *wc-2*, *ccg-9*) had a similar expression pattern but also demonstrated differences (Figure 2B). In the co-culture/crossed condition, seven circadian clock-related gene homologs exhibited a nearly unified expression pattern characterized by a highest peak and similar expression level (Figure 2C). Additionally, given that the *MAT1-1*, *MAT1-2* and their co-culture/crossed conditions potentially have different rhythmic periods for circadian gene homologs (Figure 2A–C), further studies are needed.

### 3.5. Expression Patterns of Four Mating-Type Genes in MAT1-1, MAT1-2 and MAT1-1 × MAT1-2 Co-Culture

To elucidate the rhythmic periodicity of four mating-type genes, we applied RT-qPCR and assessed the expression patterns of *MAT1-1-1*, *MAT1-1-10*, and *MAT1-1-11* in the *MAT1-1* strain, *MAT1-2-1* in the *MAT1-2* strain, and *MAT1-1-1*, *MAT1-1-10*, *MAT1-1-11,* and *MAT1-2-1* in the co-culture/crossed condition at the conidiation stage at eight time points during the ZT cycle of 24 h.

#### 3.5.1. Expression Patterns of Three Mating-Type Genes in the *MAT1-1* Strain

**Figure 3:**
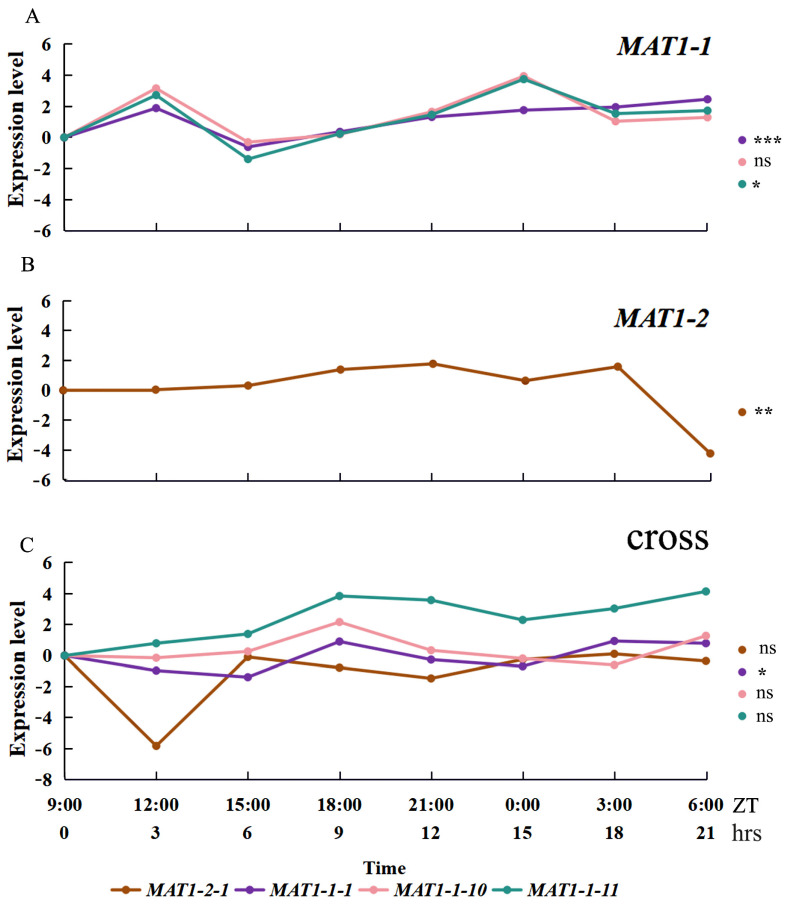
Rhythmical oscillations in gene expression of four mating-type genes (*MAT1-1-1*, *MAT1-2-1*, *MAT1-1-10* and *MAT1-1-11*) of *M. sextelata* in its *MAT1-1* (**A**), *MAT1-2* (**B**) and *MAT1-1* × *MAT1-2* co-culture/crossed condition (**C**) strains. Total RNA was isolated from each strain and harvested every 3 h for one day (x-axis). The expression levels of the four mating-type genes were quantified using the expression of the housekeeping *actin1* as a reference (y-axis). Each dot represents the mean of three biological replicates. Note that the transcript profiles of the four mating-type genes (except *MAT1-2-1*) oscillate in generally similar patterns in each strain, but are totally different among the three strains, reaching peak levels at different timepoints. Zeitgeber time (ZT) is depicted. Statistical significance was assessed by one-way ANOVA (*: *p* < 0.05; **: *p* < 0.01; ***: *p* < 0.001; ns = not significant).

In the *MAT1-1* strain (Figure 3A), both *MAT1-1-10* and *MAT1-1-11* had the same rhythmic periodicity from ZT9:00 to ZT6:00, exhibiting expression peaks at both ZT12:00 and ZT0:00 and expression troughs at both ZT15:00 and ZT3:00 with a nearly identical expression level (Figure 3A). From ZT9:00 to ZT21:00, *MAT1-1-1* showed a similar rhythmic periodicity as both *MAT1-1-10* and *MAT1-1-11*, with one expression peak at ZT12:00, but *MAT1-1-1* had a very different expression pattern from the other two genes after ZT21:00. According to one-way ANOVA, *MAT1-1-1* and *MAT1-1-11* displayed significant rhythmic expression changes (Figure 3A), whereas *MAT1-1-10* showed no statistically significant rhythmicity. Notably, despite lacking significance, *MAT1-1-10* exhibited a rhythmic peak similar to that of *MAT1-1-11*, implying a coordinated expression program between the two genes.

#### 3.5.2. Expression Patterns of One Mating-Type Gene in the *MAT1-2* Strain

In the *MAT1-2* strain, the expression pattern of *MAT1-2-1* (Figure 3B) was totally different from those of *MAT1-1-1*, *MAT1-1-10* and *MAT1-1-11* in the *MAT1-1* strain (Figure 3A). The gene *MAT1-2-1* did not show a regular fluctuation periodicity from ZT9:00 to ZT6:00 and exhibited a steep trough at ZT6:00. One-way ANOVA analysis revealed significant rhythmic expression changes in *MAT1-2-1* (Figure 3B).

#### 3.5.3. Expression Patterns of Four Mating-Type Genes in the *MAT1-1* × *MAT1-2* Co-Culture

In the co-culture/crossed condition, the expression pattern of *MAT1-1-1*, *MAT1-1-10*, *MAT1-1-11* and *MAT1-2-1* (Figure 3C) was totally different from those in the *MAT1-1* and *MAT1-2* strains (Figure 3A,B). Interestingly, the expression of *MAT1-1-10* and *MAT1-1-11* still showed a consistent rhythmic pattern in the cross, remaining different from that of *MAT1-1-1*. Moreover, the expression of *MAT1-2-1* in the co-culture/crossed condition exhibited an opposite pattern to that in the *MAT1-2* strain, showing the lowest trough at ZT12:00. According to one-way ANOVA analysis, only *MAT1-1-1* among the four mating-type genes showed significant rhythmic expression changes, while *MAT1-1-10*, *MAT1-1-11* and *MAT1-2-1* did not exhibit statistically significant rhythmicity (Figure 3C). *MAT1-1-10* and *MAT1-1-11* exhibited similar rhythms to that of *MAT1-1-1*.

#### 3.5.4. Comparison of Expression Patterns Among *MAT1-1*, *MAT1-2* and *MAT1-1* × *MAT1-2* Co-Culture

On the one hand, the expression pattern of *MAT1-1-1*, *MAT1-1-10*, *MAT1-1-11* and *MAT1-2-1* in the co-culture/crossed condition was totally different from those in the *MAT1-1* and *MAT1-2* strains (Figure 3). On the other hand, *MAT1-1-10* and *MAT1-1-11* in the *MAT1-1* strain exhibited relatively regular circadian fluctuations, indicating a rhythm periodicity of 12 h. However, *MAT1-1-1* in the *MAT1-1* strain, *MAT1-2-1* in the *MAT1-2* strain, and *MAT1-1-1*, *MAT1-1-10*, *MAT1-1-11*, and *MAT1-2-1* in the co-culture/crossed condition did not show clear regular circadian fluctuations and their rhythm periodicity is currently unknown, indicating further research with a longer duration needed.

### 3.6. Comparison of Eleven Gene Expression Patterns Among MAT1-1, MAT1-2 and MAT1-1 × MAT1-2 Co-Culture

**Figure 4:**
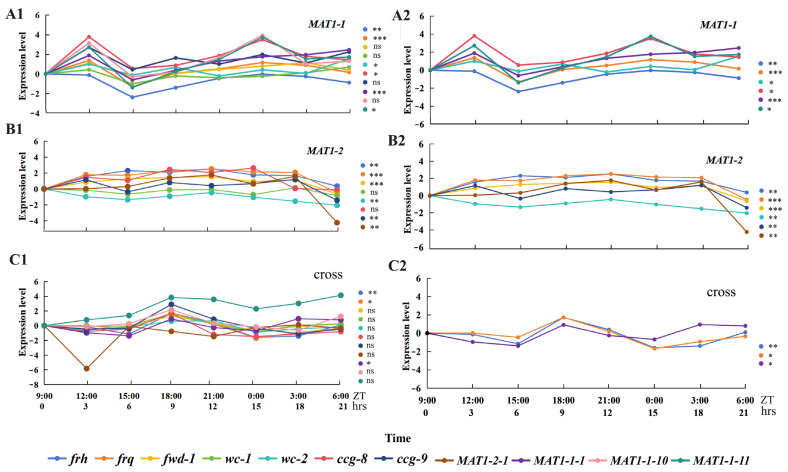
Comparison of rhythmic oscillations of gene expression between seven clock homologous genes (*frh*, *frq*, *fwd-1*, *wc-1*, *wc-2*, *ccg-8* and *ccg-9*) and four mating-type genes (*MAT1-1-1*, *MAT1-2-1*, *MAT1-1-10* and *MAT1-1-11*) of *M. sextelata* in its *MAT1-1* ((**A1**), all genes; (**A2**), significant genes), *MAT1-2* ((**B1**), all genes; (**B2**), significant genes) and *MAT1-1* × *MAT1-2* co-culture/crossed condition ((**C1**), all genes; (**C2**), significant genes). Total RNA was isolated from each strain and harvested every 3 h for one day (x-axis). The expression levels of the seven homologous genes and four mating-type genes were quantified using the expression of the housekeeping *act1* as a reference (y-axis). Each dot represents the mean of three biological replicates. Note that the transcript profiles of eleven genes oscillate in generally similar patterns in each strain, except *MAT1-2-1* and *MAT1-1-11* in the *MAT1-1* × *MAT1-2* co-culture/crossed condition, but are totally different among the three strains, reaching peak levels at different timepoints. Zeitgeber time (ZT) is depicted. *frh*: frequency-interacting RNA helicase; *frq*: frequency; *wc*: white collar; *ccg*: clock-controlled gene. Statistical significance was assessed by one-way ANOVA (*: *p* < 0.05; **: *p* < 0.01; ***: *p* < 0.001; ns = not significant).

To investigate whether the four mating-type genes exhibited circadian expression patterns similar to the seven circadian clock homologs, we analyzed their expression profiles within each strain, including either all investigated genes (Figure 4(A1,B1,C1)) or only significantly rhythmic genes (Figure 4(A2,B2,C2)). In both the *MAT1-1* strain (Figure 4(A1,A2)) and *MAT1-2* strain (Figure 4 (B1,B2)), the mating-type genes and their corresponding clock homologs displayed remarkably consistent expression patterns and rhythmic fluctuations. In the co-culture/crossed condition, these genes also showed generally consistent expression dynamics (Figure 4(C1,C2)), except for *MAT1-2-1* (non-significant), which exhibited a distinct low trough at ZT12:00 (Figure 4(C1)). Interestingly, as core components of the fungal circadian clock system, the expression rhythms of *frh* and *frq* were significant across the *MAT1-1*, *MAT1-2* and their co-culture/crossed strains, and *MAT1-1-1* showed significant rhythms in both *MAT1-1* and crossed strains. In *M. sextelata*, the expression rhythms of these eleven genes were highly conserved within each strain but varied consistently across the three strains, regardless of whether all investigated genes or only significantly rhythmic ones were considered. Together, these results support the existence of an endogenous circadian clock governing conidiation in *M. sextelata*, although its rhythms appear to differ among mating-type strains and their co-culture/crossed condition strains.

## 4. Discussions

Among fungi, the ascomycete *N. crassa* stands as the sole model system in which the molecular underpinnings of circadian rhythms have been extensively characterized, especially during conidiation [11,36,37]. However, the molecular architecture of circadian clocks in other fungal species remains largely unknown. In this study, we investigated the circadian components of *M. sextelata*, which has been successfully cultivated on a commercial scale in China [24,38], and further evaluated their rhythmic expression patterns in *MAT1-1*, *MAT1-2*, and their *MAT1-1* × *MAT1-2* co-culture/crossed condition during conidiation. Our results demonstrate that *M. sextelata* harbors core components of the circadian oscillator, exhibiting rhythmic expression for eleven genes examined. Notably, distinct expression patterns and peak times were observed among the three investigated conditions, which represent novel characteristics distinct from those previously reported in other fungi [37,39,40].

The core circadian oscillator is organized around a feedback loop governed by the FRQ, FRH, WC-1, WC-2, and FWD-1 in *N. crassa*, and controls other clock-controlled genes (*ccgs*) in a circadian fashion [39,41,42]. To date, all fungal species in which the *frq* gene has been identified possess a circadian clock [19,43]. However, not all fungi with circadian rhythmicity harbor the *frq* gene [32,44]. In comparison with *frq*, *wc-1* and *wc-2* are widely conserved in the fungi [22,41]. In this study, through homologous sequence alignment and bioinformatic analyses, we identified seven homologs in the *M. sextelata* genome that exhibit high sequence similarity to circadian clock genes characterized in *N. crassa*, including the core ones *frq*, *frh*, *wc-1*, *wc-2*, and *fwd-1*, as well as *ccg-8* and *ccg-9*. The results indicate that *M. sextelata* carries the conserved components required for FWC oscillator operationality as previously reported in *N. crassa*, and possesses the inherent ability to manifest circadian rhythmicity.

Then, the expression patterns of seven circadian homologs and four mating-type genes were assessed via RT-qPCR in this study, showing a surprisingly nearly consistent expression pattern and rhythm fluctuation within each of the three strains but uniformly different ones among the three strains, except for the gene *MAT1-2-1* in the co-culture/crossed condition (Figure 2, Figure 3 and Figure 4). In the *MAT1-1* strain, all genes (seven clock genes, *MAT1-1-1*, *MAT1-1-10* and *MAT1-1-11*) exhibited a transcriptional peak at ZT12:00 (the middle of the day), with most also showing a secondary peak at ZT0:00 (dawn time). In contrast, these genes (seven clock genes and *MAT1-2-1*) lacked distinct sharp peaks in the *MAT1-2* strain, displaying only a broad expression peak spanning ZT9:00 to ZT6:00 throughout the experimental period. Notably, in the co-culture/crossed condition, all genes (seven clock genes, *MAT1-1-1*, *MAT1-1-10*, *MAT1-1-11* and *MAT1-2-1*) showed a clear peak at ZT18:00 (dusk time) except *MAT1-2-1*, totally different from those observed in either the *MAT1-1* or *MAT1-2* strain. In *N. crassa*, the expression of its clock genes exhibits only a single peak within a 24 h period [5,22,36,39], similar to our finding of only one peak in the co-culture/crossed condition of *M. sextelata* (Figure 2C). In contrast, the *MAT1-1* strain of *M. sextelata* displays multiple expression peaks, whereas the *MAT1-2* strain yields a single broad, gently rounded peak (Figure 2A,B).

In addition, the peak time of most known *ccgs* in *N. crassa* largely coincides with that of the core clock gene *frq*, occurring from late night to early morning (dawn time), although they differ in overall expression levels and rhythmic amplitude, with only a few *ccgs* peaking at midday (noon time) or night (dusk time) [45,46]. These distinct expression phases of circadian genes are also observed in mice with transcriptional “rush hours” at dawn and dusk [47]. However, in *M. sextelata*, the peak time of the investigated clock genes and mating-type genes occurred at noon and dawn time in the *MAT1-1* strain, but at dusk time in the co-culture/crossed condition, without an obvious peak time observed in the *MAT1-2* strain. As previous circadian clock studies did not consider the mating types of the strains used [45,46,48], whether the expression peak numbers and peak times arise from differences in mating type or species remains unclear and warrants further investigation. However, the degree to which conidiation is governed by the circadian clock in the fungal kingdom suggests a selective advantage for initiating these developmental processes at specific times of the day [10,11].

The key to the mating process in heterothallic fungi is communication between cells of the opposite mating-type [29,49,50]. To investigate whether strain communications and interactions have potential influence on the expression patterns of circadian clock genes when different mating-type strains are co-cultured/crossed, we sought to assess the daily oscillations of clock genes and mating-type gene expression in the co-culture/crossed condition under the same culture conditions as the *MAT1-1* and *MAT1-2* strains. Remarkably, in contrast to the parental strains, circadian clock genes and mating type genes in the co-culture/crossed condition exhibited highly different but uniform expression patterns, characterized by a shared phase and coordinated fluctuations (Figure 4(A1–C2)). In addition, phenotypic observations revealed that the growth characteristics of the co-culture/crossed condition also differ significantly from either the *MAT1-1* or *MAT1-2* strains, which displayed only sporadically scattered conidia on their respective media, while the co-culture/crossed condition had the conidia clustered densely in the contacted region between *MAT1-1* and *MAT1-2* (Figure 1). These divergences suggest that potential direct or indirect interaction between the *MAT1-1* and *MAT1-2* regulatory systems reorganizes circadian control in the co-culture/crossed condition, thereby further coordinating reproductive processes. Although stronger conidiation was evident at the contact region, heterokaryon formation could not be directly validated. Accordingly, the disparate transcriptional patterns observed in co-cultures are tentatively attributed to interactions between distinct *MAT* strains instead of serving as direct proof of heterokaryon-exclusive circadian regulation.

Notably, *MAT1-1-1*, *MAT1-1-10*, and *MAT1-1-11* displayed expression phases and fluctuation patterns nearly consistent with those of core circadian clock genes in both the *MAT1-1* strain and the co-culture/crossed condition, suggesting that these mating-type genes may be associated, either directly or indirectly, with clock-related regulation in *M. sextelata*. Whereas expression oscillations of *MAT1-2-1* reached statistical significance in the *MAT1-2* strain but not in the co-culture/crossed condition, its expression dynamics also diverged from those of core circadian clock genes, suggesting that this mating-type gene might lack circadian-associated rhythmicity in *M. sextelata*. The circadian clock provides an endogenous signal to regulate conidiation on a daily basis in *N. crassa* [36,46,51]. Since the expression peaks of the *MAT1-1-1*, *MAT1-1-10*, and *MAT1-1-11* genes in *M. sextelata* are roughly consistent with those of the core clock homologous genes, we hypothesize that these mating-type genes might be involved in conidial development.

Regarding RT-qPCR normalization, the stability of candidate reference genes, *actin1*, *elongation factor 1-α* and *β-tubulin* were compared using BestKeeper based on their raw Ct values, and all three candidates showed acceptable stability (SD < 1). *Actin1* showed the lowest SD value and a high correlation with the BestKeeper index. Although *elongation factor 1-α* had a slightly higher correlation coefficient, *actin1* exhibited lower Ct variation and a moderate expression level. Accordingly, *actin1* was selected to normalize relative gene expression throughout the circadian time of *M. sextelata*, as this gene has been also adopted for circadian research on model fungi exemplified by *Saccharomyces cerevisiae*.

## 5. Conclusions

In summary, the present study demonstrates that the core circadian components are evolutionarily conserved in *M. sextelata* and exhibit distinct rhythmic expression patterns. These findings enrich the evolutionary framework of fungal chronobiology, and provide a new perspective for investigating the potential association between the circadian clock and mating-type regulation, thereby further advancing the current understanding of circadian regulatory mechanisms in fungi. Because stable and efficient genetic manipulation systems for *Morchella* species remain technically challenging, reverse genetics studies should be performed once such systems become available to elucidate the precise functions of core clock genes. Future studies should incorporate a larger number of strains with different mating types and their corresponding co-culture/crossed combinations to verify the relationship between circadian oscillations and mating type background. Moreover, manipulating circadian clock genes holds potential value for optimizing the growth and fruiting body production of *Morchella*. Further exploration of these strategies would benefit the efficient cultivation and industrial application of this important edible fungus in the food industry.

## Figures and Tables

**Table 1:** Gene homology comparison in *M. sextelata* using BLASTX searches based on 17 circadian clock genes identified from *N. crassa*, *A. chevalieri*, *A. melleus* and *Lachnellula hyalina.* “/” means the gene is unmatched in the genome of *M. sextelata*. E value, identity and coverage were used to evaluate sequence similarity between *M. sextelata* candidates and the corresponding query proteins. Putative homologs were selected using E value < 1 × 10^−10^, identity > 30%, and coverage ≥ 25% as the main criteria. Seven finally chosen homologous genes used in this study for assessing rhythmic oscillations in *M. sextelata* are indicated in bold.

Gene ID	Gene Name	Accession Number	Species	E Value	Identity (%)	Coverage (%)
H6S33_001260	***frh***	NC_026502.1	*Neurospora crassa*	0	61.04	72
H6S33_012933	***frq***	NC_026507.1	*N. crassa*	4 × 10^−25^	30.85	36
H6S33_010463	***fwd-1***	NC_026507.1	*N. crassa*	4 × 10^−93^	54.28	25
H6S33_012964	***wc-1***	NC_057368.1	*Aspergillus chevalieri*	2 × 10^−150^	45.21	64
H6S33_003590	***wc-2***	NW_025763372.1	*A. mellus*	6 × 10^−31^	38.26	57
H6S33_003804	***ccg-8***	NW_022157136.1	*Lachnellula hyalina*	2 × 10^−70^	38.74	73
H6S33_008721	***ccg-9***	NC_026507.1	*N. crassa*	5 × 10^−163^	42.62	44
H6S33_008775	*ccg-15*	NC_026505.1	*N. crassa*	1 × 10^−43^	33.33	24
H6S33_005012	*ccg-16*	NC_026506.1	*N. crassa*	6 × 10^−13^	51.72	21
H6S33_009409	*ccg-14*	NC_026505.1	*N. crassa*	2 × 10^−30^	50	12
/	*ccg-1*	NC_026505.1	*N. crassa*	/	/	/
/	*ccg-4*	NW_022157138.1	*L. hyalina*	/	/	/
/	*ccg-6*	NC_026505.1	*N. crassa*	/	/	/
/	*ccg-13*	NC_026505.1	*N. crassa*	/	/	/
/	*mfa-1*	NC_026505.1	*N. crassa*	/	/	/
/	*pre-1*	NC_026503.1	*N. crassa*	/	/	/
/	*pre-2*	NC_026507.1	*N. crassa*	/	/	/

**Table 2:** Primers used for amplifying the reference gene, seven clock homologous genes and four mating-type genes by RT-qPCR, along with their corresponding product sizes.

Gene ID	Gene Name	Primer Sequence (5′→3′)	Amplicon Size (bp)
Reference gene	*actin1*	Forward: GTACCCTGGTATTGCCGACC	197
		Reverse: GGACGATGGAAGGACCACTC	
H6S33_001260	*frh*	Forward: TCCTTTGCACAACTCTCCAC	174
		Reverse: GCGATGAATCCAAGACGTCT	
H6S33_012933	*frq*	Forward: AACACACATACCACCACACC	150
		Reverse: CCGTGATGTGTAGGTTGAGG	
H6S33_010463	*fwd-1*	Forward: TCTGGAATCGAGAGCAGCTA	147
		Reverse: AGGTTGCTTGCTGGTTGTTA	
H6S33_012964	*wc-1*	Forward: AGCGACATTGTACCGGTTAC	192
		Reverse: CAACTTGTAAACCGGCCTCT	
H6S33_003590	*wc-2*	Forward: GGAACATCAGCAACAACAGC	161
		Reverse: GTGTAGGTTTTGCAGGTGGA	
H6S33_003804	*ccg-8*	Forward: ATATGATGCAGCAGGTGAGC	166
		Reverse: TCCTCCAACCCCTCAAATCT	
H6S33_008721	*ccg-9*	Forward: CTTCTGCGTACACACTCTCC	178
		Reverse: GCTCCATAGAAACGAAGGCA	
NA *	*MAT1-1-1*	Forward: CCCACCTTCTGAGTCCGT	143
		Reverse: GAACCCAGATTCCACCGC	
H6S33_000236 *	*MAT1-2-1*	Forward: GCCAGAAATACCCGGGGAT	135
		Reverse: GCTTTACGTGGTGCTCTCG	
NA *	*MAT1-1-10*	Forward: CGATGAGCTTGGGGATGC	137
		Reverse: GATGCAGACACCCTTGGG	
NA *	*MAT1-1-11*	Forward: CGTTAGATGAGGCGACGC	107
		Reverse: CTCAACCTGTCGTGCTGC	

* NA: not available. The online genome (GenBank accession number: GCA_020137385.1) of the *M. sextelata* strain contains only *MAT1-2-1*. The sequences of *MAT1-1-1*, *MAT1-1-10*, and *MAT1-1-11* were obtained via PCR amplication.

**Table 3:** BestKeeper-based stability analysis of candidate reference genes for RT-qPCR normalization.

Candidate Reference Gene	Mean Ct	SD	CV (%)	Correlation with BestKeeper Index (r)	Stability Ranking/Decision
*A* *ctin1*	22.69	0.80	3.52	0.925	Lowest SD; selected as internal reference
*Elongation factor 1-α*	21.64	0.88	4.08	0.926	Stable, but not selected
*β-tubulin*	29.07	0.89	3.05	0.846	Stable, but relatively low expression and lower correlation

## Data Availability

The data supporting the findings of this study are included within the article. Further inquiries can be directed to the corresponding author.
